# Supplementary material for: Plasma-state metasurfaces for ultra-intensive field manipulation
Source: Light Sci Appl. 2026 Jul 9;15:307. doi: 10.1038/s41377-026-02304-7 (PMC13350927; doi:10.1038/s41377-026-02304-7)
Supplement: Supplementary file 1 — Supplementary Material [file 41377_2026_2304_MOESM1_ESM.pdf]

Supplementary Information

Plasma-state metasurfaces for ultra-intensive field manipulation

Zi-Yu Chen*, Hao Xu, Jiao Jia, Yanjie Chen, Siyu Chen, Yan Zhang, Mingxuan Wei, Minghao Ma, Runze Li, Fan Yang, Mo Li, Guangwei Lu, Weijun Zhou, Hanmi Mou, Zhuofan Zhang, Zhida Yang, Jian Gao, Feng liu, Boyuan Li, Min Chen, Liming Chen, Yongtian Wang, Lingling Huang*, Wenchao Yan*, Shuang Zhang*, Jie Zhang*

*Corresponding author. E-mail: ziyuch@scu.edu.cn; huanglingling@bit.edu.cn; wenchaoyan@sjtu.edu.cn; shuzhang@hku.hk; jzhang1@sjtu.edu.cn

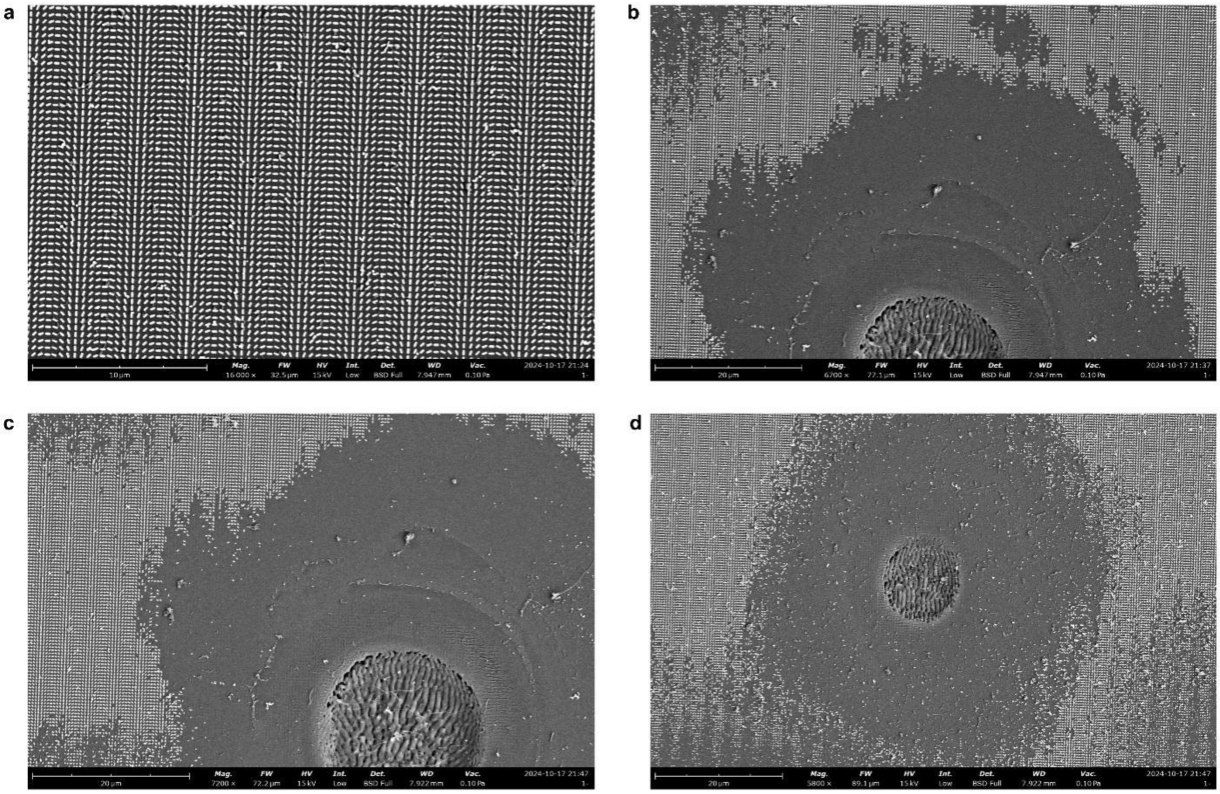

Fig. S1. Magnified scanning electron microscopy (SEM) images of the metasurfaces. a, SEM image of the linear phase-gradient PB metasurfaces before the interaction. **b-d,** SEM images of the metasurfaces after the intense laser irradiation in different regions.

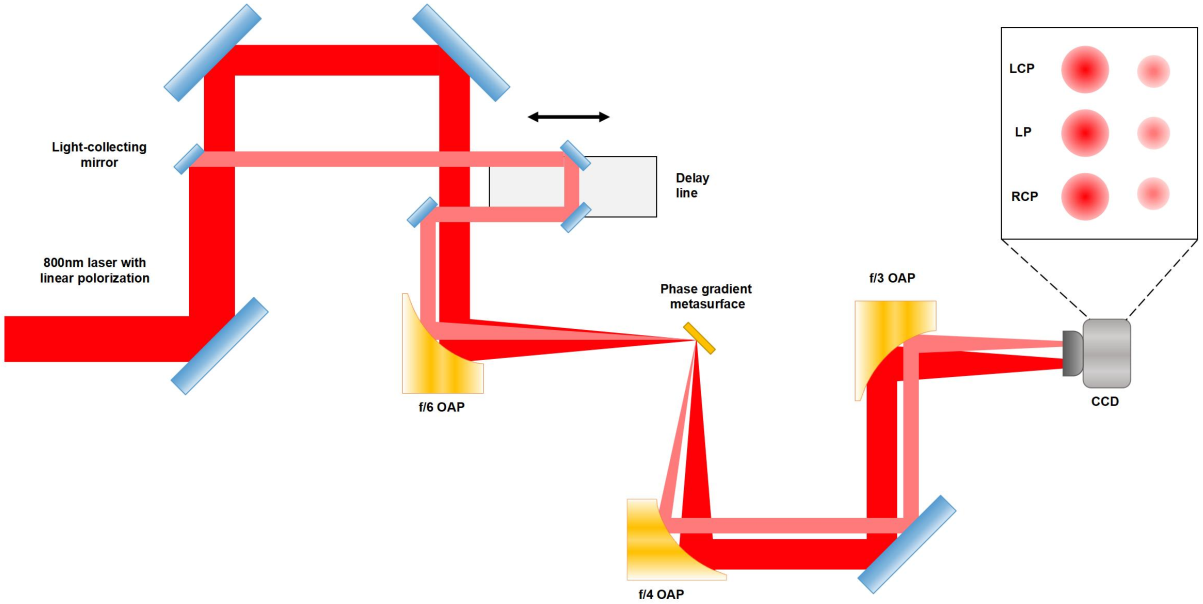

Fig. S2. Experimental setup schematic for measuring the operational lifetime of the plasma metasurfaces. A small portion of the main laser pulse is split as a probe beam. The reflected intensities of the probe beam are recorded as the time delay between the pump and probe beams is varied. The metasurfaces have the same structure with those used in the photonic spin Hall effect experiments.

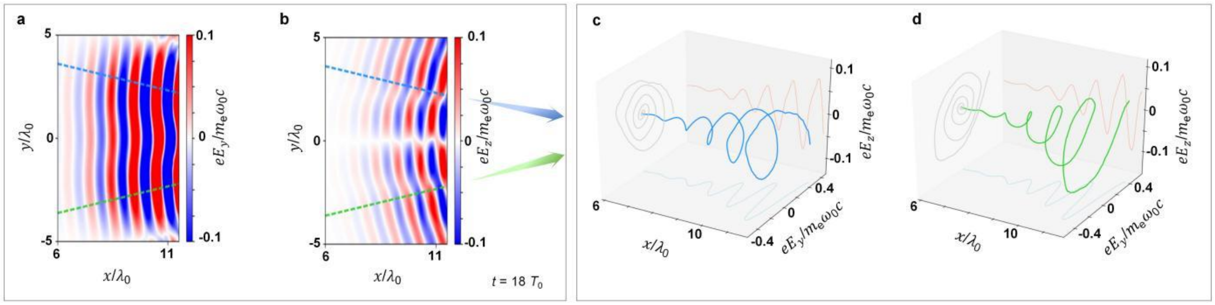

Fig. S3. 3D PIC simulation results of the photonic spin Hall effect with considering preplasma effects. a, b, 2D electric filed profiles in the x-y plane at $t = 18T_0$ after the reflection for the (**a**) E_y and (**b**) E_z components of the reflected pulse, respectively. Blue and green dashed lines indicate the anomalous reflection directions induced by the metasurfaces. **c, d,** Reconstructed 3D electric field vector images along the anomalous reflection directions in the (**c**) upper and (**d**) lower half spaces, respectively. Here, a preplasma with an exponential density gradient profile $n_\mathrm{e}(x) = n_0 \exp((x - x_0)/L_\mathrm{s})$ is introduced in front of the meta-atom slab, where $x_0 = 20\lambda_0$ represents the front surface of the plasma slab and $L_\mathrm{s} = 0.1\lambda_0$ is the preplasma scale length.

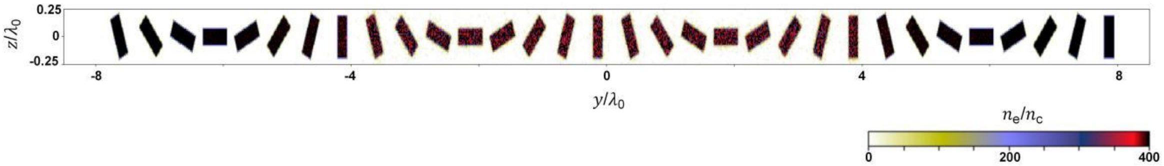

Fig. S4. Plasma electron density distribution from 3D PIC simulations. Normalized Electron density profile in the *y*-*z* plane at $t = 18T_0$ which is after the laser reflection from the plasmas. The initial electron density is $n_\mathrm{e} = 400n_\mathrm{c}$. The target is composed of fully ionized plasmas, i.e., free electrons and ions, where ions are assumed as immobile due to ultrashort timescales.

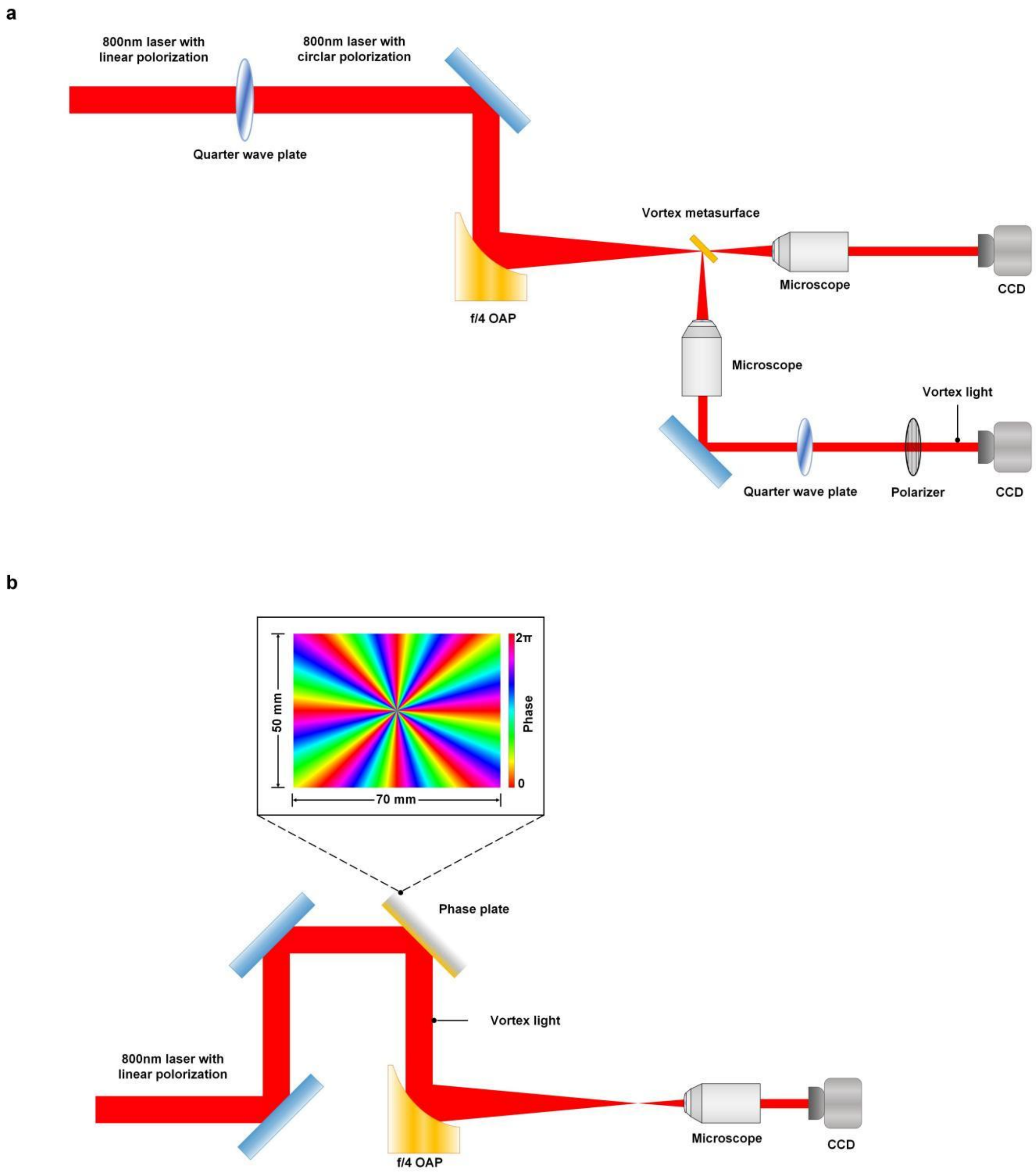

Fig. S5. Experimental setup schematic for optical vortex generation. a, Experimental setup for measuring optical vortices generated by metasurfaces. **b,** Schematic diagram of the experimental setup for optical vortices generated by spiral phase mirrors. The inset shows the phase distribution of the spiral phase mirror.

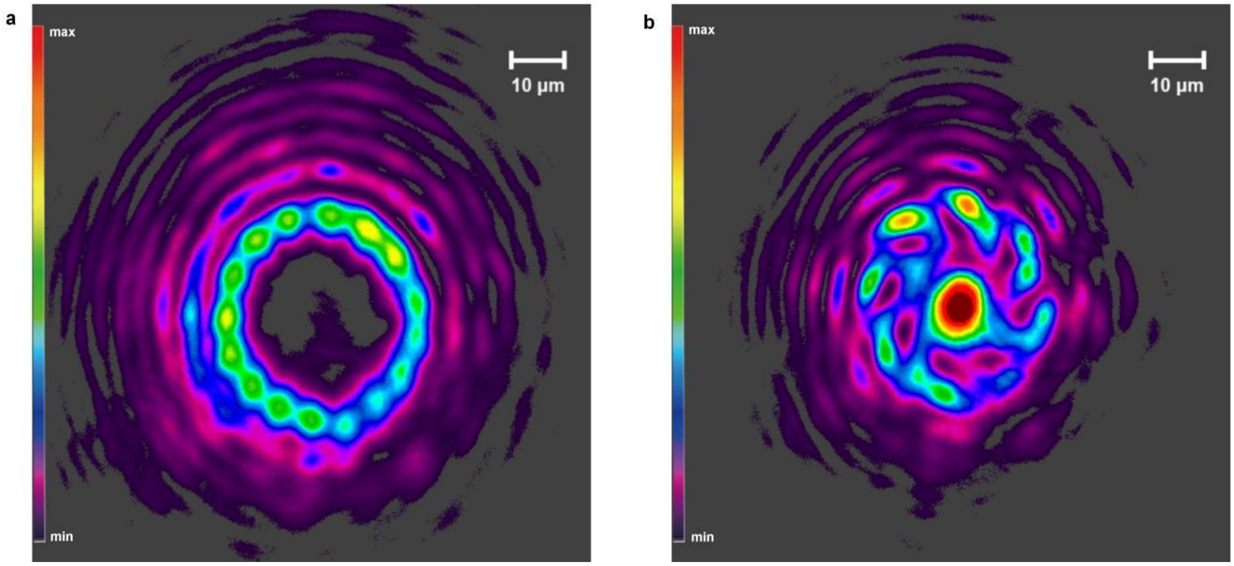

Fig. S6. Experimental results of optical vortex generation from solid-state metasurfaces. a, Measured intensity profile of the vortex beam generated from the solid-state metasurface sample at low laser intensity. **b,** Interference intensity profile between the PB-phase-affected vortex pulse and the unaffected Gaussian pulse from the solid-state metasurfaces, implying a topological charge of $l = 8$. The interference is measured with the linear polarizer rotated 60° relative to its orientation in panel **a**.

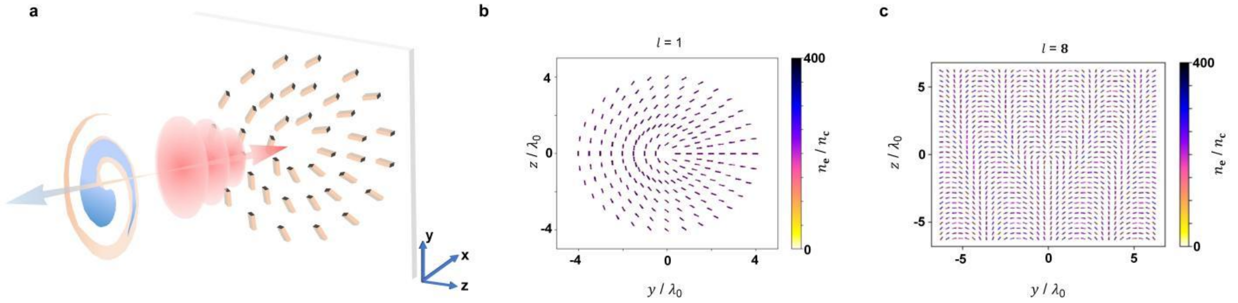

Fig. S7. 3D PIC simulation results of optical vortex generation from plasma-state metasurfaces in the relativistic regime. a, A schematic diagram of the vortex beam generation. A circularly polarized (CP) Gaussian laser pulse with an intensity of 9.6×10^{18} W cm^{-2} is incident onto the plasma-state metasurfaces with density of $n_\mathrm{e} = 400 n_\mathrm{c}$. The laser incidence angle is 0° and 14.5° for the $l = 1$ and $l = 8$ case, respectively. The reflected pulse contains optical vortices carrying an orbital angular momentum. **b, c,** The electron density distribution of the plasma-state metasurface target with a topological charge of (**b**) $l = 1$ and (**c**) $l = 8$.

Supplementary note 1: plasma metasurfaces assisted efficient particle acceleration

Laser-driven plasma-based acceleration, which is capable of supporting high acceleration gradients, has shown considerable promise for revolutionizing areas such as the generation of high-quality particles and novel light sources[1-4]. Among the various acceleration schemes, the interaction of high-intensity lasers with solid-density plasmas is especially important for ion acceleration, the production of high-charge energetic electrons, bright x-ray generation, and the creation of extreme energy density conditions[5], etc. The key challenge in laser-solid plasma interactions is efficiently transferring energy from the laser to the plasma. This is important for the aforementioned applications. One approach to improve energy transfer is to use grating targets. These targets can excite surface plasmons[6], which can enhance the acceleration of protons and directional electrons along the target surface[7-9]. However, this only works at specific laser angles. Here we show metasurfaces can be used to improve the efficiency of laser-plasma interactions. By carefully designing the metasurface, it is possible to reduce the dependence on specific resonance angles and further enhance energy coupling. This can lead to more efficient particle acceleration and other applications. According to conservation of momentum in the parallel direction to the target surface, we have $k_{\parallel}^{\mathrm{r}} = k_{\parallel}^{\mathrm{in}} + \xi$, where ξ is the phase gradient of the metasurfaces. This gives the generalized Snell's law of reflection[10]: $\sin\theta^{\mathrm{r}} = \sin\theta^{\mathrm{in}} + \xi/k_0$. When the phase gradient is larger than the incident wave number, i.e., $\xi > k_0$, the excitation of surface plasmons occurs even at normal incidence with $\theta^{in} = 0^{\circ}$[11]. This means metasurfaces enable efficient energy coupling at a broad range of incident angles, which simplify the experimental setup and make it easier to control the interaction. We simulate linear phase-gradient metasurfaces with a period of $3.3\lambda_0$, corresponding to a critical incident angle of $\theta^{\mathrm{in}} = 45^{\circ}$. We show such plasma-state metasurfaces (not optimized) already lead to significantly higher particle yields and cutoff energies compared to conventional plane targets (Supplementary Fig. 8, 9). The greatly enhanced directional energetic electrons along the target surface and high-energy protons have important implications for applications such as cancer therapies.

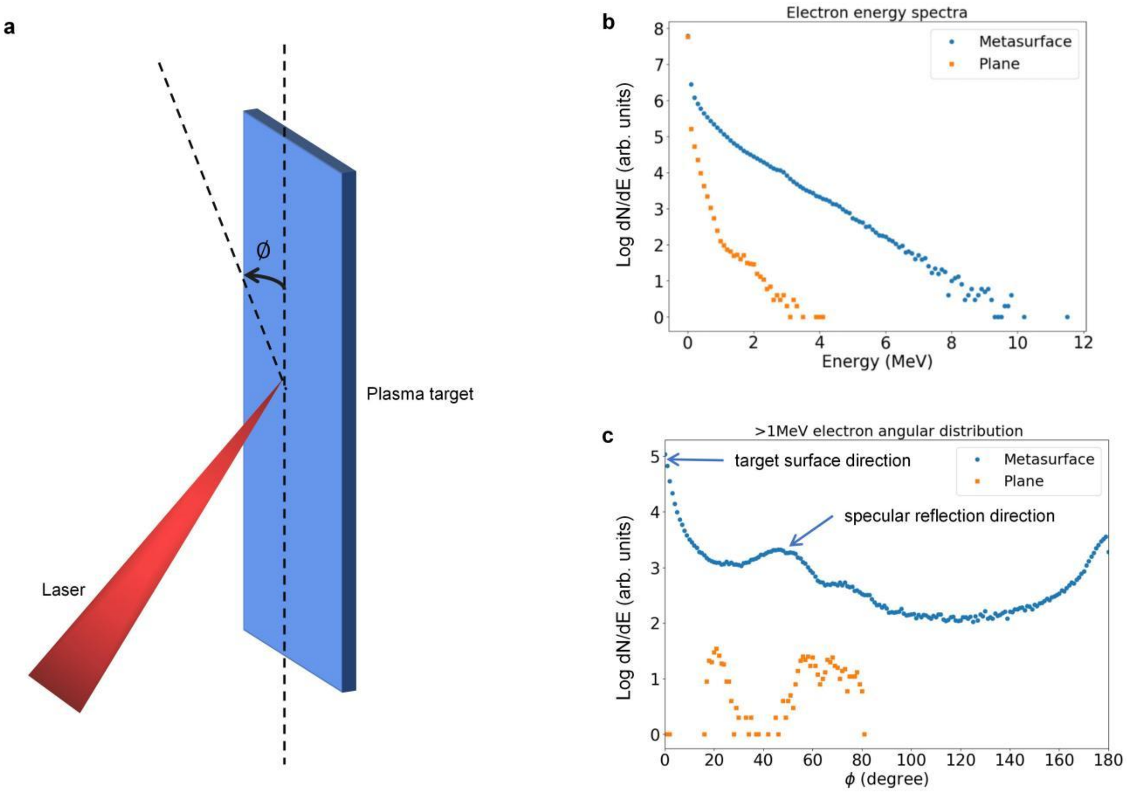

Fig. S8. 3D PIC simulation results of laser electron acceleration by relativistic surface plasmons from plane and metasurface plasma-targets. a, Scheme of the laser electron acceleration. A circularly polarized relativistic laser is obliquely incident onto the plasma target. The laser normalized amplitude is $a_0 = 3$, corresponding to an intensity of 3.8×10^{19} W cm^{-2}. The plasma density is $n_{\mathrm{e}} = 70 n_{\mathrm{c}}$. The oblique incidence angle is $\theta = 50^{\circ}$. The linear phase-gradient metasurface consists of eight cuboids within a single unit cell, with each cuboid spaced $0.4\lambda_0$ (320 nm) apart in the y-direction. **b,** Comparison of electron energy spectra generated by plasma-state metasurface targets versus plane targets. **c,** Comparison of electron (energy >1 MeV) angular distribution generated by metasurface plasma-targets versus plane targets. The yield of accelerated electrons from metasurface plasma-target is significantly higher than that from plane target in all directions, especially along the target surface direction.

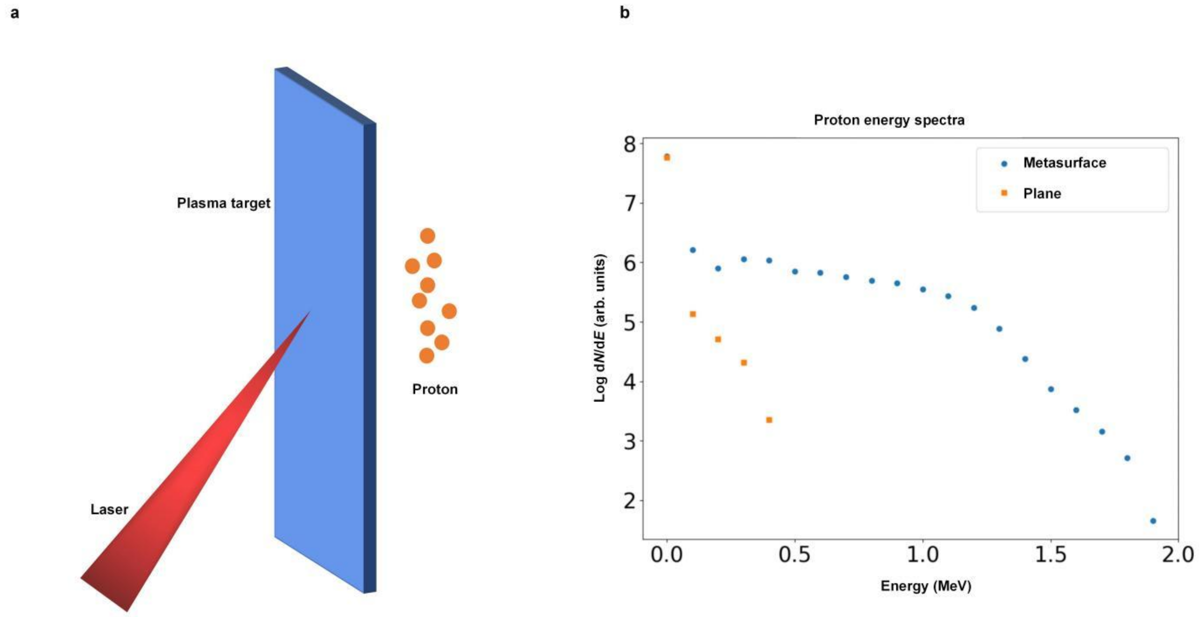

Fig. S9. 3D PIC simulation results of laser proton acceleration by target sheath normal acceleration from plane and metasurface plasma-targets. a, Scheme of the laser proton acceleration. A circularly polarized relativistic laser is obliquely incident onto the plasma target. The laser normalized amplitude is $a_0 = 3$, corresponding to an intensity of 3.8×10^{19} W cm^{-2}. The plasma density is $n_\mathrm{e} = 70 n_\mathrm{c}$. The oblique incidence angle is $\theta = 50^\circ$. The linear phase-gradient metasurface consists of eight cuboids within a single unit cell, with each cuboid spaced $0.4\lambda_0$ (320 nm) apart in the *y*-direction. **b,** Comparison of proton energy spectra generated by metasurface plasma-targets versus plane targets. The cutoff energy of accelerated energetic protons from metasurface plasma-target is increased by a factor of 4 compared to that from plane plasma-target.

References

1. Esarey, E., Schroeder, C. B., & Leemans, W. Physics of laser-driven plasma-based electron accelerators. *Rev. Mod. Phys.* **81**, 1229 (2009).

2. Macchi, A., Borghesi, M. & Passoni, M. Ion acceleration by superintense laser-plasma interaction. *Rev. Mod. Phys*. **85**, 751 (2013).

3. Corde, S., Ta Phuoc, K., Lambert, G., Fitour, R., Malka, V., & Rousse, A. Femtosecond x rays from laser-plasma accelerators. *Rev. Mod. Phys*. **85**, 1 (2013).

4. Teubner, U. & Gibbon, P. High-order harmonics from laser-irradiated plasma surfaces. *Rev. Mod. Phys.* **81**, 445 (2009).

5. Beier, N. F. et al. Homogeneous, Micron-Scale High-Energy-Density Matter Generated by Relativistic Laser-Solid Interactions. *Phys.Rev.Lett.* **129**, 135001 (2022).

6. Macchi, A. et al. Extreme high field plasmonics Electron acceleration and XUV harmonic generation from ultrashort surface plasmons. *Phys.Plasmas* **26**, 042114 (2019).

7. Ceccotti, T. et al. Evidence of resonant surface-wave excitation in the relativistic regime through measurements of proton acceleration from grating targets. *Phys. Rev. Lett.* **111**, 185001 (2013).

8. Fedeli, L. et al. Electron acceleration by relativistic surface plasmons in laser-grating interaction. *Phys. Rev. Lett.* **116**, 015001 (2016).

9. Cantono, G. et al. Extreme ultraviolet beam enhancement by relativistic surface plasmons. *Phys. Rev. Lett.* **120**, 264803 (2018).

10. Yu, N. et al. Light propagation with phase discontinuities: generalized laws of reflection and refraction. *Science* **334**, 333–337 (2011).

11. Sun, S. et al. Gradient-index meta-surfaces as a bridge linking propagating waves and surface waves. *Nat. Mater*. **11**, 426–431 (2012).
